# Gill slits provide a window into the respiratory physiology of sharks

**DOI:** 10.1093/conphys/coaa102

**Published:** 2020-12-04

**Authors:** Wade J VanderWright, Jennifer S Bigman, Cayley F Elcombe, Nicholas K Dulvy

**Affiliations:** Earth to Ocean Research Group, Department of Biological Sciences, Simon Fraser University, 8888 University Drive, Burnaby, British Columbia, V5A 1S6, Canada

**Keywords:** *Carcharhinus*, Gill Oxygen Limitation Theory, Metabolic Theory of Ecology, *Rhizoprionodon terraenovae*, shrinking fishes, *Sphyrna tiburo*

## Abstract

Metabolically important traits, such as gill surface area and metabolic rate, underpin life histories, population dynamics and extinction risk, as they govern the availability of energy for growth, survival and reproduction. Estimating both gill surface area and metabolic rate can be challenging, especially when working with large-bodied, threatened species. Ideally, these traits, and respiratory physiology in general, could be inferred from external morphology using a faster, non-lethal method. Gill slit height is quick to measure on live organisms and is anatomically connected to the gill arch. Here, we relate gill slit height and gill surface area for five Carcharhiniform sharks. We compared both total and parabranchial gill surface area to mean and individual gill slit height in physical specimens. We also compared empirical measurements of relative gill slit height (i.e. in proportion to total length) to those estimated from field guide illustrations to examine the potential of using anatomical drawings to measure gill slit height. We find strong positive relationships between gill slit height and gill surface area at two scales: (i) for total gill surface area and mean gill slit height across species and (ii) for parabranchial gill surface area and individual gill slit height within and across species. We also find that gill slit height is a consistent proportion of the fork length of physical specimens. Consequently, relative gill slit height measured from field guide illustrations proved to be surprisingly comparable to those measured from physical specimens. While the generality of our findings needs to be evaluated across a wider range of taxonomy and ecological lifestyles, they offer the opportunity that we might only need to go to the library and measure field guide illustrations to yield a non-lethal, first-order approximation of the respiratory physiology of sharks.

## Teaser

Metabolic traits—such as metabolic rate and gill surface area—likely underlie life histories, population dynamics and extinction risk. Yet, measuring these traits requires lethal sampling and is time-intensive. We find that measures of gill slit height are positively related to estimates of gill surface area in five shark species.

## Introduction

Many exploited fishes are data-poor, lacking the demographic and life history data for fisheries assessments (Sadovy, 2005; Ricard *et al.*, 2012; Temple *et al.*, 2020). This is especially true for chondrichthyans (sharks, rays and chimaeras, hereafter ‘sharks’) as 46% of all species are categorized as Data Deficient on the IUCN Red List (IUCN Red List of Threatened Species, 2020). The interconnection between life histories, vulnerability to overfishing and population decline is increasingly well understood (Dulvy and Reynolds, 2002; Field *et al.*, 2009; Dulvy *et al.*, 2014; Juan-Jordá *et al.*, 2015). Larger-bodied species with slower growth rates are more likely to decline as a result of overfishing than their smaller, faster-growing relatives. However, estimating time-related life history traits, such as growth, age at maturity and longevity, still requires time-intensive lethal sampling of many (50+) individuals, and even then, age estimation is fraught with challenges (Pardo *et al.*, 2013; Smart *et al.*, 2013; Harry, 2018). Our working thesis is that simple morphological traits (i.e. heritable characteristics) that relate to metabolic rate and gill surface area—such as gill slit height—may offer other time-related traits with which to infer the response of species to threats, such as overfishing, and the effects of elevated temperature and lower oxygen availability from climate change (Chown *et al.*, 2004; Pauly, 2010).

Metabolism provides a unique bridge from organismal physiology to the life histories and ecology of populations. Metabolic rate governs the rate of resource uptake and allocation and therefore underlies trade-offs in organismal survival, growth and reproduction (Hennemann, 1983; Gillooly *et al.*, 2002; Brown *et al.*, 2004). Measuring metabolic rate typically requires a laboratory setting or a highly controlled field setting, which is often difficult to implement for larger-bodied, more active species (Carlson *et al.*, 2004; Byrnes *et al.*, 2020). Consequently, there are few (3%; 35 out of 1200) published metabolic rate estimates for sharks (Bouyoucos *et al.*, 2019; Lyons *et al.*, 2019). However, the connection between morphology and metabolism is becoming increasingly apparent and facilitates examining respiratory physiology on longer, integrated time scales beyond acute (Wegner, 2011; Killen *et al.*, 2016; Bigman *et al.*, 2018). For example, respiratory surface area—or gill surface area in aquatic ectotherms—and metabolic rate are tightly correlated and scale at the same rate with body mass both intraspecifically within species and interspecifically across species (De Jager and Dekkers, 1975; Gillooly *et al.*, 2016). On an organismal level, experimental reductions in gill surface area via ablation result in concomitant reductions in metabolic scope (Duthie and Hughes, 1987). Because estimating gill surface area requires lethal sampling and the measurement process is highly time-consuming (it typically takes 30–40 h for one individual to estimate gill surface area for a single specimen), there is a need for an easy-to-measure, non-lethal morphological correlate of metabolic rate and gill surface area.

**Figure 1:**
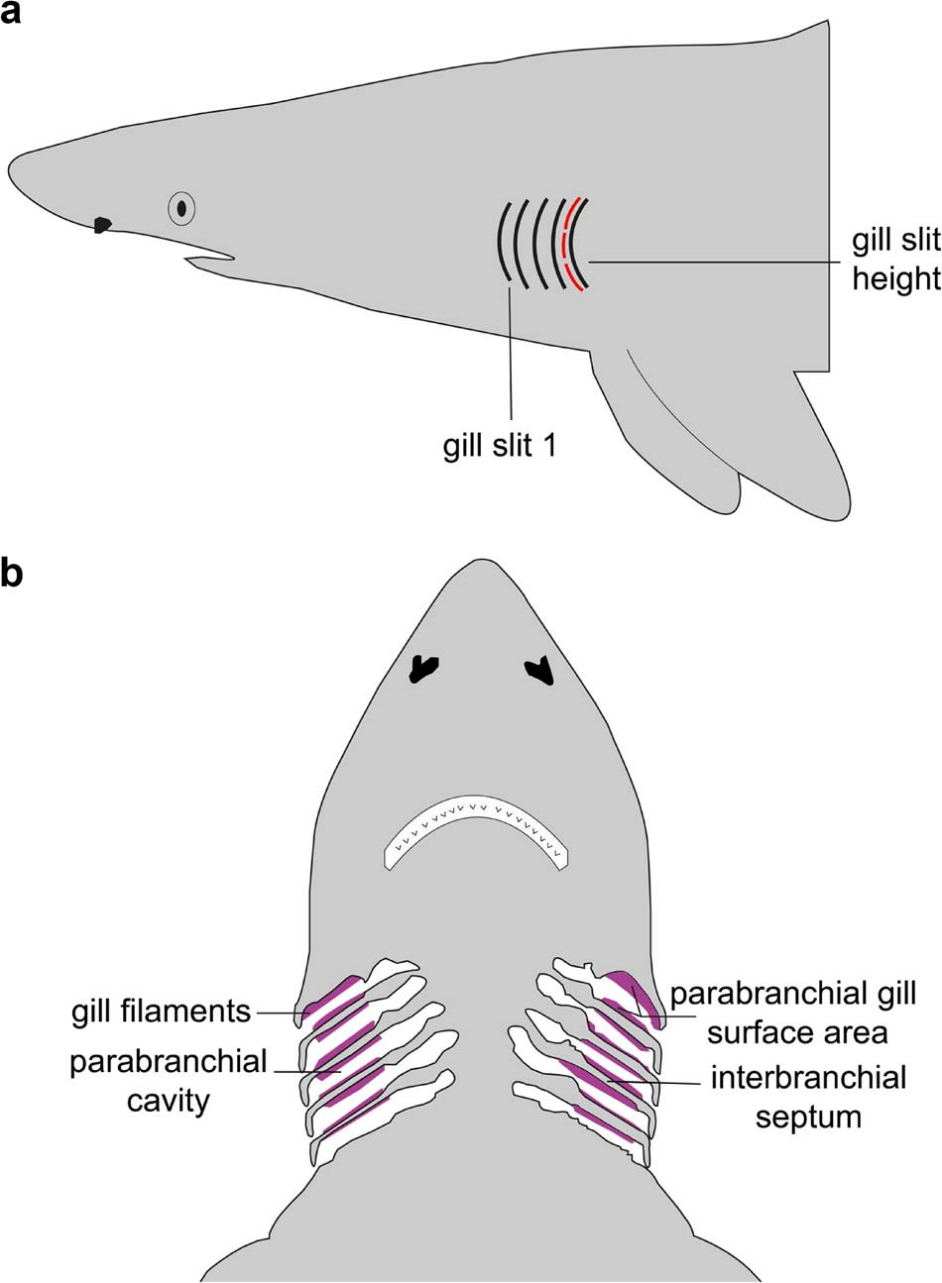
(**a**) The general external gill slit morphology of Carcharhiniformes. The red dashed line depicts the height of an individual gill slit. (**b**) Diagram of the internal respiratory morphology of Carcharhiniformes

Gill slit height measurements are an attractive alternative to measuring gill surface area and metabolic rate as they can be rapidly measured from live animals in the field, eliminating the need to sacrifice and transport specimens. In sharks, the gill slit is the external extension of the interbranchial septum, the structure that separates and supports the gill arches (Fig. 1; Wegner, 2011). Most sharks have five gill slits on each side of the head that correspond to five parabranchial cavities (Fig. 1). Each parabranchial cavity houses hemibranchs of gill filaments and lamellae—the surface area where oxygen uptake occurs (Fig. 1). Hence, there is a direct morphological connection between the surface area of the gills and height of the gill slit opening (Wegner, 2011, 2015). Since gill surface area is limited by the cross-sectional area of the head, and thus the size of the parabranchial cavities, it follows that gill slit height would be under a similar morphological limitation (Wegner, 2011, 2015; Wootton *et al.*, 2015). Thus, total gill surface area (i.e. the gill surface area of all parabranchial cavities on both sides of the head) may vary predictably with mean gill slit height (i.e. the mean height of all individual gill slits). This pattern is likely mirrored at a finer scale; the parabranchial gill surface area, or the gill surface area within each parabranchial cavity (i.e. the space between each interbranchial septum through which water flows during ventilation; Fig. 1b), may be related to the height of the corresponding gill slit (i.e. the individual gill slit height).

 In addition to gill slit height measurements from physical specimens, gill slit heights can be measured from field guide illustrations. Morphological measurements from such illustrations are increasingly used in meta-analytical research of fish and other vertebrates, such as the relationships between bird plumage colour and sexual selection (Dale *et al.*, 2015) and caudal fin aspect ratios and activity level in fish (Palomares and Pauly, 1989; Bigman *et al.*, 2018).

Here, we assess the relationship of gill surface area and gill slit height in five Carcharhiniform shark species. In addition, we compare measurements of gill slit height from physical (i.e. field-collected) specimens to those from field guide illustrations to identify if they are a promising avenue for non-lethal sampling. We asked the following three questions: (i) does the scaling of total gill surface area and mean gill slit height differ across species, (ii) does the scaling of parabranchial gill surface area and individual gill slit height differ within and across species and (iii) are the measurements of relative gill slit height (i.e. mean gill slit height in proportion to fork length) from field guide illustrations consistent with those from physical specimens?

## Methods

We first describe the methods for collection and preservation of specimens. Second, we describe how both gill surface area and gill slit height were measured. Third, we explain how the anatomical measurements were extracted from field guide illustrations and compared to measurements from physical specimens, and finally, we detail our statistical methods. Animal Care Committee permits #1128B-14 and #1273B-14 were obtained from Simon Fraser University for this research.

### Collection and preservation of specimens

All five gill arches from both sides of the head were retained from 46 individuals of five Carcharhiniform species collected opportunistically from fisheries-independent and fisheries-dependent gill net and longline surveys in the Western Central Atlantic Ocean and Gulf of Mexico (Blacknose Shark *Carcharhinus acronotus n* = 8, Blacktip Shark *Carcharhinus limbatus n* = 9, Bonnethead Shark *Sphyrna tiburo n* = 9, Finetooth Shark *Carcharhinus isodon n* = 10 and Atlantic Sharpnose Shark *Rhizoprionodon terraenovae n* = 10)*.* Fork length (FL cm; length of snout to fork in caudal fin) was recorded for each specimen upon landing (Blacknose Shark 40.5–95 cm FL, Blacktip Shark 45.5–133 cm FL, Bonnethead Shark 39–89.5 cm FL, Finetooth Shark 45–120 cm FL and Atlantic Sharpnose Shark 30–78.5 cm FL). To minimize shrinkage, gills were placed in 10% neutral-buffered formalin upon collection (Wootton *et al.*, 2015).

### Gill surface area measurement

Total gill surface area was estimated using dissection and microscopy following Muir and Hughes (1969) and Hughes (1984) as detailed in Bigman *et al*. (2018). Briefly, total gill surface area (*A*) is equal to

$$A={L}_{\mathrm{fil}}\ast 2{n}_{\mathrm{lam}}\ast{A}_{\mathrm{lam}},$$

where ${L}_{\mathrm{fil}}$ is the total length of all gill filaments (filament length), ${n}_{\mathrm{lam}}$ is the average number of lamellae per unit length on one side of a filament (lamellar frequency) and ${A}_{\mathrm{lam}}$ is the mean bilateral surface area of a lamella (bilateral lamellar surface area). Because both sides of the head are assumed to be symmetrical, total gill surface area is estimated from measurements of filament length, lamellar frequency and bilateral lamellar surface area on one side of the head (e.g. see Wegner, 2011; Wootton *et al.*, 2015; Bigman *et al.*, 2018). Parabranchial gill surface area was estimated as above, with the exception that ${L}_{\mathrm{fil}}$ equals the length of all the gill filaments in the respective parabranchial cavity on one side of the head. The lamellar frequency (${n}_{\mathrm{lam}}$) and the bilateral lamellar surface area (${A}_{\mathrm{lam}}$) remained the same as above.

### Gill slit height measurement on physical specimens

Individual gill slit height on one side of the head, or both sides of the head if possible, was measured to the nearest millimetre using a flexible measuring tape following Dolce and Wilga (2013). The anterior-most gill slit was designated as ‘gill slit 1’, and subsequent arches were successively numbered toward the posterior of the animal. The tape was lain flat along each gill slit and then stretched from the ventral end of the gill slit toward the dorsal end, following the natural curvature of each gill slit (red line in Fig. 1a). Individual gill slit height was measured three times and averaged for each specimen. The mean gill slit height was then calculated by taking the average of all five individual gill slit heights. For most specimens (*n* = 40 of 46), gill slit height was measured on both sides of the head. It was determined that both sides were symmetrical, as the average difference in mean gill slit height between the left and right sides for all individuals was 3.1% and a two-sample *t*-test showed no significant difference between mean gill slit height from the left and right sides (*t* = −0.233, d.f. = 78, *P* value = 0.8164).

### Gill slit height measurement on field guide illustrations

Field guide illustrations were taken from *Sharks of the World*, a comprehensive and widely used field guide for all sharks and one where a sole scientific illustrator drew all illustrations (Ebert *et al.*, 2013). We specifically chose this field guide as it contains all known shark species at the time of publishing, which is crucial if morphological measurements taken from field guide illustrations will prove to be a reliable source in lieu of physical specimens. Images were cropped for maximum pixel accuracy using Adobe Photoshop CC (2018, version 19.1.8). Image-processing software (ImageJ, NIH) was used to take measurements of fork length (length of snout to fork in caudal fin) and gill slit height on each illustration (one illustration per species).

### Comparison of gill slit heights between physical specimens and field guide illustrations

To assess if gill slit height measured from field guide illustrations shows promise in being a reliable estimate of gill slit height for physical specimens, we compared measurements of relative gill slit height from physical specimens to those of field guide illustrations for the same five Carcharhiniform species using 95% confidence intervals (95% CI) of relative gill slit height for each species. First, we estimated relative gill slit height on physical specimens by taking a mean of all gill slit heights on one individual (mean gill slit height) and then dividing this number by the fork length of the individual. This produced a measurement of the relative gill slit height, a dimensionless ratio. Second, we calculated the relative gill slit height on each field guide illustration in the same manner: the gill slit heights were averaged for each illustration and then divided by the fork length (snout to fork in the caudal fin) of each illustration. Third, we estimated a bootstrapped 95% CI of the relative gill slit heights from physical specimens. To do so, we bootstrapped 100 estimates of relative gill slit height for each species from a normal distribution with a mean and standard deviation estimated from the species-specific relative gill slit heights measured on physical specimens. The relative gill slit height from field guide illustrations and physical specimens was considered to be different if the relative gill slit height from field guide illustrations was not included in the bootstrapped 95% CI of the relative gill slit height estimated from physical specimens. We also computed the Pearson correlation coefficient, which measures the linear correlation between two variables (Zuur *et al.*, 2009).

### Statistical analyses

All data were log_10_-transformed prior to analyses in order to estimate power law allometric scaling relationships on a linear scale, log_10_(*y*) = log_10_(*a*) + *b* * log_10_(*x*), where *x* and *y* are the respective response and predictor variables (i.e. for the scaling relationship of total gill surface area and mean gill slit height, total gill surface area = *y* and mean gill slit height = *x*), *a* is the intercept (i.e. the *y*-variable at *x* = 1) and *b* is the slope of the scaling relationship.

Simple linear regression was used to estimate coefficients for all scaling relationships, both within and across species as implemented by lm function in R v3.6.1. (R Core Team, 2013; RStudio Team, 2015). For both within- and across-species analyses, we used simple linear models with the R-language formula notation, ‘log_10_(*y*) ~ log_10_(*x*) * species’, where a separate slope and intercept were estimated for each species. To assess differences across species, mean slope or intercept values for each species were compared to the 95% CI of the slopes and intercepts of the other species (e.g. Gillooly *et al.*, 2016; Bigman *et al.*, 2018).

## Results

### Does the scaling of total gill surface area and mean gill slit height differ across species?

**Figure 2:**
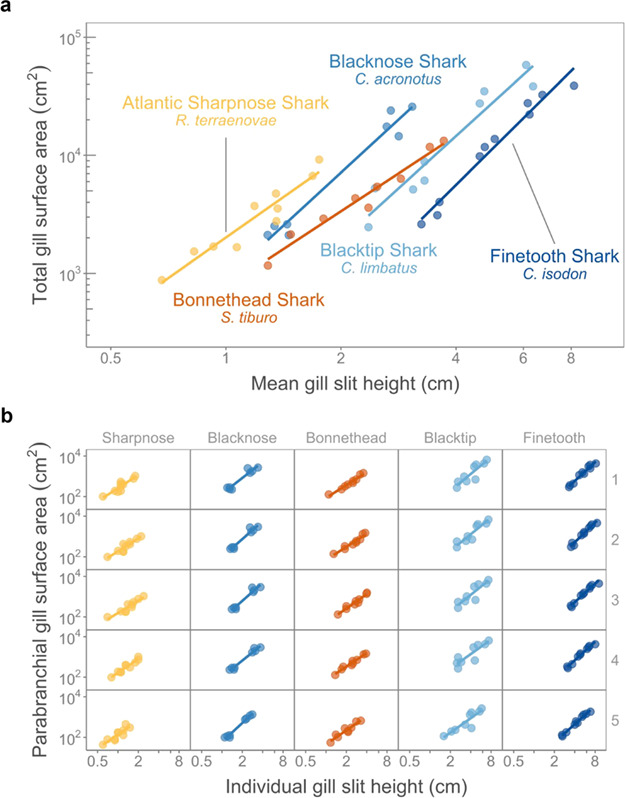
The total gill surface area (cm^2^) and mean gill slit height (cm) (**a**) across five Carcharhiniformes species and (**b**) across each of the five gill slits within each species. Note that the numbers to the right side of the y-axis indicate which gill slit and parabranchial cavity are plotted in each row

The relationship of total gill surface area and mean gill slit height was largely consistent across species (Fig. 2a). The 95% CI of the slope of total gill surface area and mean gill slit height overlapped for all five species (Fig. 2a, Table 1). The shallowest slope observed was that from the Bonnethead Shark (2.13, 95% CI = 0.99–3.27), and the Finetooth Shark had the steepest slope (3.21, 95% CI = 2.03–4.39; Fig. 2a, Table 1).

**Table 1:** The relationship of total gill surface area and mean gill slit height for five shark species

**Species**	**Intercept (cm^2^)**	**Intercept 95% CI**	**Slope**	**Slope 95% CI**
Blacknose Shark *Carcharhinus acronotus*	903.15	628.46–1297.91	2.98	2.51–3.46
Finetooth Shark *Carcharhinus isodon*	65.84	17.99–240.97	3.21	2.03–4.39
Blacktip Shark *Carcharhinus limbatus*	244.86	81.75–733.41	2.95	1.81–4.08
Atlantic Sharpnose Shark *Rhizoprionodon terraenovae*	2011.34	936.48–4319.90	2.28	1.10–3.46
Bonnethead Shark *Sphyrna tiburo*	768.29	307.27–1920.97	2.13	0.99–3.27

All intercept values and their 95% confidence intervals (CI) are back-transformed.

### Does the scaling of parabranchial gill surface area and individual gill slit height differ within and across species?

**Table 2:** The relationship of parabranchial gill surface area and individual gill slit height for five shark species

**Species**	**Intercept (cm^2^)**	**Intercept 95% CI**	**Slope**	**Slope 95% CI**
**Parabranchial cavity 1**				
Blacknose Shark *Carcharhinus acronotus*	135.69	84.91–216.84	2.61	1.99–3.23
Finetooth Shark *Carcharhinus isodon*	7.98	1.34–47.59	3.10	1.51–4.69
Blacktip Shark *Carcharhinus limbatus*	28.22	5.96–133.62	2.76	1.22–4.31
Atlantic Sharpnose Shark *Rhizoprionodon terraenovae*	260.48	96.56–702.68	2.16	0.56–3.76
Bonnethead Shark *Sphyrna tiburo*	85.17	24.89–291.47	2.12	0.57–3.67
**Parabranchial cavity 2**				
Blacknose Shark *Carcharhinus acronotus*	98.32	62.75–154.05	2.93	2.37–3.50
Finetooth Shark *Carcharhinus isodon*	5.98	1.18–30.21	3.22	1.81–4.63
Blacktip Shark *Carcharhinus limbatus*	30.16	7.86–115.67	2.71	1.37–4.04
Atlantic Sharpnose Shark *Rhizoprionodon terraenovae*	186.68	71.52–487.24	2.12	0.74–3.51
Bonnethead Shark *Sphyrna tiburo*	69.80	21.49–226.69	2.15	0.77–3.53
**Parabranchial cavity 3**				
Blacknose Shark *Carcharhinus acronotus*	80.01	47.00–136.21	2.96	2.34–3.58
Finetooth Shark *Carcharhinus isodon*	7.99	1.33–48.15	3.03	1.49–4.57
Blacktip Shark *Carcharhinus limbatus*	29.60	6.20–141.43	2.69	1.21–4.18
Atlantic Sharpnose Shark *Rhizoprionodon terraenovae*	156.46	49.76–491.92	2.04	0.53–3.55
Bonnethead Shark *Sphyrna tiburo*	51.67	12.54–212.84	2.34	0.79–3.89
**Parabranchial cavity 4**				
Blacknose Shark *Carcharhinus acronotus*	102.62	63.89–164.82	2.69	2.13–3.25
Finetooth Shark *Carcharhinus isodon*	10.11	1.98–51.54	3.03	1.62–4.45
Blacktip Shark *Carcharhinus limbatus*	50.39	12.73–199.46	2.39	1.06–3.72
Atlantic Sharpnose Shark *Rhizoprionodon terraenovae*	167.03	59.95–465.36	2.25	0.81–3.70
Bonnethead Shark *Sphyrna tiburo*	71.16	20.58–246.02	2.23	0.81–3.64
**Parabranchial cavity 5**				
Blacknose Shark *Carcharhinus acronotus*	60.91	39.53–93.83	3.08	2.43–3.74
Finetooth Shark *Carcharhinus isodon*	7.03	1.57–31.36	3.00	1.41–4.59
Blacktip Shark *Carcharhinus limbatus*	33.31	9.58–115.90	2.36	0.86–3.87
Atlantic Sharpnose Shark *Rhizoprionodon terraenovae*	139.31	55.89–347.25	2.28	0.60–3.97
Bonnethead Shark *Sphyrna tiburo*	45.63	14.93–139.43	2.30	0.67–3.92

All intercept values and their 95% confidence intervals (CI) are back-transformed.

The rate of increase in parabranchial gill surface area with individual gill slit for each cavity was similar across species (Fig. 2b, Table 2). Although not significant, the Finetooth Shark generally had steeper slope values, on average, and the Atlantic Sharpnose Shark and Bonnethead Shark had shallower slope values, on average, compared to the other species (Fig. 2b, Table 2). For all species, the fifth parabranchial cavity consistently had the least gill surface area and shortest gill slit height (Fig. 2b, Table 2).

### Are the measurements of relative gill slit height from field guide illustrations consistent with those from physical specimens?

**Figure 3:**
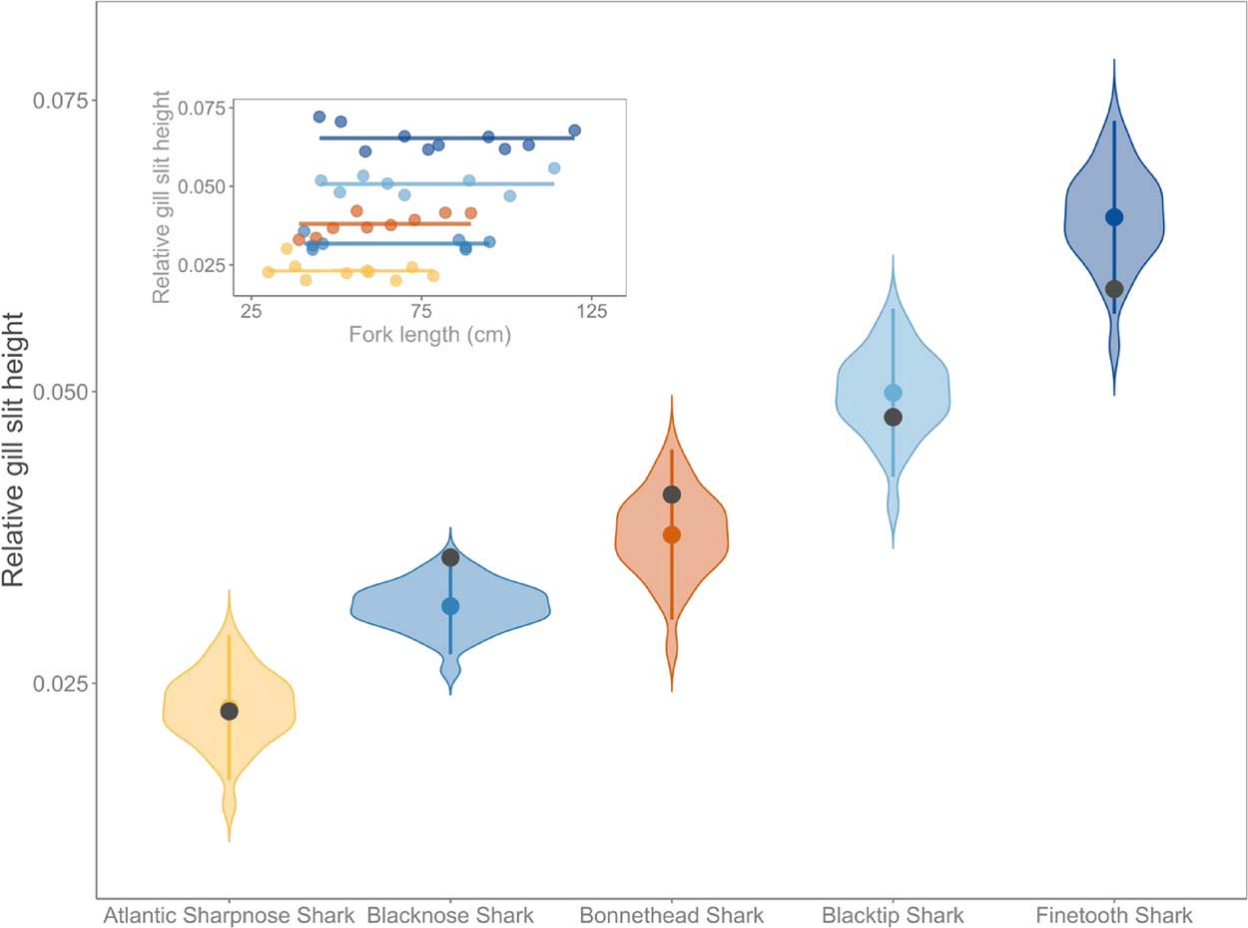
The distribution of relative gill slit heights (i.e. the proportion of mean gill slit height to fork length) for each species estimated by bootstrapping relative gill slit heights from a normal distribution with a mean and standard deviation equal to species-specific relative gill slit heights measured on physical specimens. Overlaid are the species-specific mean (coloured circles), the 95% confidence interval (point range line) from the bootstrapped estimates, and the relative gill slit height measured on field guide illustrations (grey circles). Inset shows that the relative gill slit height is nearly constant for any length of any physical specimen (data points). Fit lines represent an intercept-only linear model fit, as the slope estimate was not significantly different from zero for all species.

Relative gill slit heights from physical specimens were broadly comparable to field guide illustrations (Fig. 3). First, the species-specific relative gill slit height obtained from field guide illustrations fell within the species-specific bootstrapped 95% CI for four of the five species (Fig. 3, Table 3). We note that the one species for which the mean relative gill slit height from the field guide illustration did not fall within the 95% CI, Blacknose Shark, was only outside the 95% CI by 0.005, or 0.5% (Table 3). We further note that the relative gill slit height measurement from the field guide illustration was almost exactly equal to a measurement from a physical specimen (field guide value = 0.0358, physical specimen value = 0.0357). The Pearson correlation coefficient (PCC) revealed that relative gill slit heights measured on field guide illustrations were highly correlated with those measured from physical specimens (PCC = 0.98, where a value of 1.0 indicates a perfect correlation).

**Table 3:** Comparison of relative gill slit height from field guide illustrations and physical specimens

**Species**	**95% CI**	**Relative gill slit height from field guide illustration**
Blacknose Shark *Carcharhinus acronotus*	0.0271–0.0353	0.0358
Finetooth Shark *Carcharhinus isodon*	0.0558–0.0724	0.0588
Blacktip Shark *Carcharhinus limbatus*	0.0419–0.0564	0.0478
Atlantic Sharpnose Shark *Rhizoprionodon terraenovae*	0.0160–0.0285	0.0226
Bonnethead Shark *Sphyrna tiburo*	0.0297–0.0443	0.0412

The 95% confidence interval (CI) was estimated for each species from bootstrapped estimates of mean relative gill slit heights from the mean and standard deviation of relative gill slit height from physical specimens.

**Table 4:** Research agenda table outlining future directions

**Research question**
How does activity level and ventilation strategy influence the relationship of gill slit height and gill surface area?
Is this pattern generalizable across the phylogenetic tree of sharks and rays?
What is the relevant importance of phylogeny and ecology in shaping the relationship of gill slit height and gill surface area in sharks and rays?
Life histories are adapted to local environments. Is the invariance of the ratio of gill slit height and fork length the same across populations within the same species?
Do the gill slit height to fork length ratios vary across different illustrators for the same species? (e.g. FAO species guides versus *Sharks of the World*)
Do rays also exhibit a relationship between gill surface area and gill slit height?
Can gill slit height be used to infer the energy usage to inform life histories and extinction risk?
Is gill slit height a proxy measure for life history and therefore help us in ecological risk assessments?
Are there other morphological traits (e.g. the cross-sectional area of the head) that could help understand the geometry of gill slit height and gill surface area?
Are there any other metabolic morphological traits that could help inform ecological risk assessments?

The relative gill slit height estimated from physical specimens was found to be a nearly constant proportion of fork length for all five species, confirming that the ratio is constant irrespective of an individual’s body size (inset, Fig. 3). In other words, the ratio of gill slit height to fork length does not change as individuals grow in body size throughout their lifetime, confirming that (i) the size of the field guide illustration from which we measured gill slit height and fork length did not affect our result and (ii) that the size of specimens used as a model for the field guide illustration did not affect our result. The Atlantic Sharpnose Shark had the smallest relative gill slit height (2.3% of FL) and the Finetooth Shark had the greatest relative gill slit height (6.5% of FL).

## Discussion

Overall, we found that gill slit height is closely related to gill surface area in the five Carcharhiniform shark species and that measuring gill slit height from field guide illustrations is a promising avenue to infer gill surface area and reduce lethal sampling for a wider range of species. Specifically, the relationship of total gill surface area and mean gill slit height was consistent across all species and this pattern was mirrored for the relationship of parabranchial gill surface area and individual gill slit height, which was consistent within (i.e. across parabranchial cavities) and across species. The three species from the genus *Carcharhinus* were more similar to each other than the other two species in their rates of increase in gill surface area and gill slit height at all scales. Thus, the close relationship between gill surface area and gill slit height, at both scales, opens the door to evaluating the predictive power of this relationship and its general applicability across this phylogenetically and morphologically divergent class of fishes.

We found a surprisingly tight positive relationship between measures of relative gill slit height extracted from illustrations from a comprehensive and widely used field guide—*Sharks of the World*—and measures from physical specimens. The relative gill slit heights derived from field guide illustrations fell either within, or close to, the 95% CI of the physical specimens. In addition, because the relative gill slit heights measured on field guide illustrations largely matched those from physical specimens that had been preserved in formalin, we confirmed, as Wootton *et al.* (2015) found, that shrinkage of tissue due to formalin is insufficient to obscure the broader patterns we are interested in. We do caution, however, that this research is dependent on accurate field guide illustrations. Scientific illustrators routinely work closely with taxonomists to ensure the highest accuracy in their field guide illustrations, such as those in *Sharks of the World* and *Rays of the World* (Ebert *et al.*, 2013; Last *et al.*, 2016)*.* Tangentially, one issue in *Rays of the World* is that only the dorsal surface of the rays is shown, therefore we cannot yet extend our approach to rays using this most trusted source of images.

Our research shows that opportunities to infer life history traits and extinction risk, albeit coarse, may only require us to measure simple external morphological traits that are related to respiratory physiology. These opportunities are not limited to physical specimens. Taxonomic descriptions and museum collections may provide morphological data that may further reduce the number of animals sacrificed from field sampling. For example, taxonomists occasionally report measurements of mean or individual gill slit height in species descriptions (El Kamel *et al.*, 2009; Allen *et al.*, 2016; de Figueiredo Petean and de Carvalho, 2018). Another possible data source for gill slit height and possibly other traits is preserved specimens or rare photos from museum collections. If generalizable across species, our findings have the potential to reduce the number of animals sacrificed for estimating gill surface area. Further, the ethics and justification of lethal sampling have been called into question, especially for data-poor and threatened species (Heupel and Simpfendorfer, 2010; Hammerschlag and Sulikowski, 2011; Sloman *et al.*, 2019). Thus, it is important to re-evaluate lethal sampling practices and determine if it is possible to devise and pursue non-lethal methods of inference.

We caution that more work is needed to generalize our findings. Gill slit morphology differs among chondrichthyan taxonomic orders (Dolce and Wilga, 2013). Broadly, sharks can be grouped into categories based on body type and gill slit morphology, which correspond to methods of ventilation, physical features and habitat (Dolce and Wilga, 2013). The similarity of morphological features is likely derived from common ancestry and phylogenetic relatedness. Thus, the highly consistent relationship of gill surface area and gill slit height, whether total gill surface area, parabranchial gill surface area, mean gill slit height or individual gill slit height, was possibly due to our comparison of species from a single order. In the future, we hope to evaluate a broader taxonomic, morphological and ecological range of sharks (and rays) to obtain a general representation of the connection between gill slit height and gill surface area. In our humble opinion, once we have a more thorough understanding of how gill slit height relates to gill surface area across a larger number of more diverse species, we can then begin using it as a tool with which to predict life history traits and extinction risk. This would entail developing models that can be used to predict the gill surface area from gill slit height obtained from either physical specimens or field guide illustrations. Subsequently, this gill surface area can be used to then infer energy expenditure, life history and even connect to the broader goals of macrophysiology—predicting ecological dynamics such as biogeography from physiology (Bigman *et al.*, 2018; Healy *et al.*, 2019; Deutsch *et al.*, 2020). Finally, we chose to focus on just one field guide because Ebert *et al*. (2013) *Sharks of the World* is the most widely used, highly cited, consistent and up-to-date field guide for all shark species*.* We are confident that these illustrations are anatomically correct as they have previously been used to derive caudal fin aspect ratios as an index of activity level (Bigman *et al.* 2018). Here, we ask the question of whether gill slit heights measured from field guide illustrations show promise in being a reliable source *in lieu* of physical specimens. We also flag that comparing field guide illustrations of the same species from different books poses a different question to the one we tackle here. This comparison of field guides is a valid endeavour and we encourage other scientists to follow it up.

We have shown here that gill slit height is a promising morphological correlate of gill surface area and metabolic physiology in general. Further, gill slit height measurements taken from other sources—such as field guide illustrations—show great promise in being used as an alternative to expensive, field-based data collection. We acknowledge that gill surface area, gill slit height, metabolic rate and traits related to life histories and population dynamics exhibit phenotypic plasticity as well as vary on evolutionary timescales across species. Indeed, discriminating between phenotypic plasticity and adaptation via selection is one of the great challenges of evolutionary biology. The incorporation of additional species with diverse physiology, ecology, as well as across a more phylogenetically diverse group of chondrichthyans will help to start teasing apart this question in the future. To that end, we lay out a research agenda that details ten questions that will help guide future directions toward generalizing our findings, examining relationships between gill slit height and gill surface area in other taxonomic groups and assessing the utility of using gill slit height as a tool to inform the response of sharks to climate change and other threats (Table 4).
